# MicroRNAs as Therapeutic Targets in Nasopharyngeal Carcinoma

**DOI:** 10.3389/fonc.2019.00756

**Published:** 2019-08-13

**Authors:** Sumei Wang, François-Xavier Claret, Wanyin Wu

**Affiliations:** ^1^Guangdong Provincial Key Laboratory of Clinical Research on Traditional Chinese Medicine Syndrome, Guangdong Provincial Hospital of Chinese Medicine, Guangzhou, China; ^2^Department of Oncology, The Second Clinical College of Guangzhou University of Chinese Medicine, Guangzhou, China; ^3^The Postdoctoral Research Station, Guangzhou University of Chinese Medicine, Guangzhou, China; ^4^Department of Systems Biology, The University of Texas MD Anderson Cancer Center, Houston, TX, United States; ^5^Experimental Therapeutic Academic Program and Cancer Biology Program, The University of Texas Graduate School of Biomedical Sciences at Houston, Houston, TX, United States

**Keywords:** microRNA, Nasopharyngeal Carcinoma (NPC), therapeutic target, biomarker, application

## Abstract

Nasopharyngeal carcinoma (NPC) is a malignancy of epithelial origin that is prone to local invasion and early distant metastasis. Although concurrent chemotherapy and radiotherapy improves the 5-year survival outcomes, persistent or recurrent disease still occurs. Therefore, novel therapeutic targets are needed for NPC patients. MicroRNAs (miRNAs) play important roles in normal cell homeostasis, and dysregulations of miRNA expression have been implicated in human cancers. In NPC, studies have revealed that miRNAs are dysregulated and involved in tumorigenesis, metastasis, invasion, resistance to chemo- and radiotherapy, and other disease- and treatment-related processes. The advantage of miRNA-based treatment approaches is that miRNAs can concurrently target multiple effectors of pathways involved in tumor cell differentiation and proliferation. Thus, miRNA-based cancer treatments, alone or combined with standard chemotherapy and/or radiotherapy, hold promise to improve treatment response and cure rates. In this review, we will summarize the dysregulation of miRNAs in NPC initiation, progression, and treatment as well as NPC-related signaling pathways, and we will discuss the potential applications of miRNAs as biomarkers and therapeutic targets in NPC patients. We conclude that miRNAs might be potential promising therapeutic targets in nasopharyngeal carcinoma.

## Introduction

### Nasopharyngeal Carcinoma (NPC)

Nasopharyngeal carcinoma is a non-lymphomatous squamous cell carcinoma that arises from the epithelial lining of the nasopharynx. Local invasion and early distant metastasis are common in NPC. Etiologic factors for NPC include Epstein-Barr virus (EBV) infection, genetic predisposition, and environmental factors (1, 2). It is extremely difficult to detect early because of its deep location and lack of obvious clinical signs in its early stages. Concurrent chemotherapy and radiotherapy is a standard treatment for late-stage NPC (3). Nevertheless, despite the effectiveness of concurrent chemotherapy and radiotherapy in treating NPC, local or regional failure in the form of persistent or recurrent disease occurs in some patients. Therefore, novel biomarkers and therapeutic strategies to improve treatment outcomes are urgently required for NPC patients.

### MicroRNAs (miRNAs)

MiRNAs are a class of endogenous non-coding RNA molecules that are typically 22–25 nucleotides long (4, 5). They are transcribed from intragenic or intergenic regions by RNA polymerase II into pri-miRNAs (at a length between 1 and 3 kb) (6), and further processed by the RNase III ribonucleases Drosha and DiGeorge syndrome critical region gene 8, *DGCR8*, complex in the nucleus into a hairpin intermediate pre-miRNA (consisting in a stem-loop structure of about 70 nucleotides) (7). The pre-miRNA is then transported from the nucleus to the cytoplasm by exportin 5 (8). After strand separation, the mature double-stranded miRNA, also known as the guide strand, is incorporated into an RNA-induced silencing complex (RISC), whole the passenger strand (miRNA^*^) is typically degraded. The RISC is the effector complex of the miRNA pathway and comprises miRNA, Argonaute proteins (Argonaute 1 to Argonaute 4) and other proteins. The mature strand is important for target recognition and for the incorporation of specific target mRNAs into RISC (8, 9). Each miRNA can potentially target many genes (about 500 on average), and about 60% of mRNAs have at least 1 evolutionarily conserved sequence that is believed to be targeted by miRNAs (10, 11).

**Figure 1:**
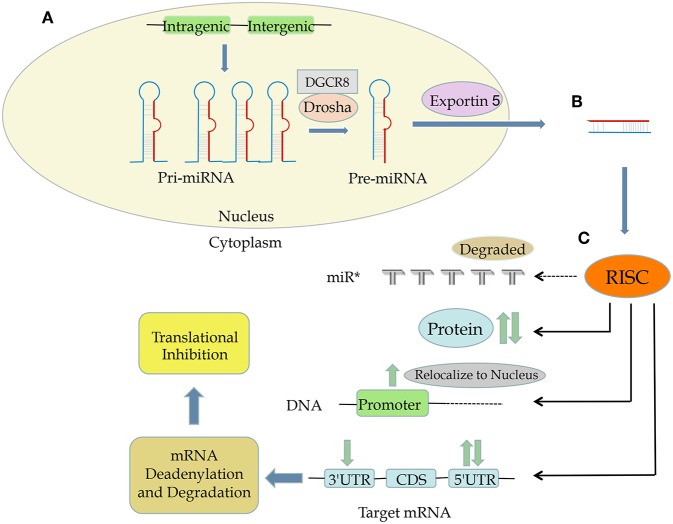
The process and functions of miRNA. **(A)** Both intergenic and intragenic genes encode miRNAs. **(B)** Mature miRNAs are transcribed from pri-miRNA and then then pre-miRNA, translocated from nucleus to cytoplasm by exportin 5, and composed of RNA-induced silencing complex (RISC). **(C)** RISC can target mRNA at 3′ UTR, CDS, and 5′ UTR as well as DNA promoter sequences and even proteins, leading to upregulation or downregulation of a specific protein.

Usually, miRNAs target the 3 prime untranslated region (3′ UTR) of their target genes, most often causing mRNA deadenylation and degradation and subsequent translational repression (5, 12). However, other miRNA-mediated mechanisms of modulating mRNA expression have also been reported. Some miRNAs bind to the open reading frame or the 5′ UTR of their target genes; in some cases, miRNAs have been shown to activate gene expression rather than suppress it (13). For example, Jopling et al. reported that miR-122 can bind to 5′ UTR, inhibiting translation of its target genes (14). In 2008, another miRNA, miR-10a, was reported to enhance translation by binding to ribosomal protein mRNA at the 5′ UTR (which is known to regulate translation) downstream of the conserved 5′TOP motif (13). In 2016, we demonstrated that miR-24 could bind to both 3′ UTR and 5′ UTR of *COPS5* (also named *JAB1* and COP9 signalosome subunit 5), leading to *COPS5* mRNA degradation and translational suppression (15). Besides, miRNAs can also regulate gene expression at the transcriptional level by binding directly to the DNA (16, 17). Moreover, proteins can also be targeted by miRNAs (18) (Figure 1).

### Exosomal miRNAs

Exosomes are microvesicles that are 40–100 nm long. They originate in intracellular endosomal compartment and are secreted by cells into their microenvironment. Exosomes transport DNA fragments, proteins, mRNAs, and miRNAs from donor cells to recipient cells and are therefore crucial to intercellular communication. Exosomal miRNAs miR-21 and miR-29a are secreted by tumor cells and can bind to toll-like receptors on nearby immune cells, thus initiating an inflammatory response that promotes metastasis (19). Furthermore, miR-21 was observed at a higher level in exosomes from the serum of patients with esophageal squamous cell carcinoma than in serum from patients with benign diseases without systemic inflammation, and an association was found between exosomal miR-21 and the presence of metastasis with inflammation (20). In addition, exosomal miR-223 was reported to be elevated in breast cancer cells and promote breast cancer invasion (21). In NPC, exosomal miR-9 was found to inhibit angiogenesis through regulating PDK/AKT pathway (22). And exosomal miR-24-3p serves as a potential biomarker for NPC prognosis (23). Therefore, exosomal miRNAs are involved in the initiation and progression of cancers including NPC, and could be biomarkers for NPC patients.

### EBV-Encoded miRNAs

EBV, a herpesvirus that infects the majority of the population worldwide asymptomatically (24), was the first human virus reported to encode miRNAs (25). More than 44 viral miRNAs are encoded from EBV. In NPC, EBV expresses EBNA1, LMP1, and LMP2A, EBERs, and BARTs (26). miR-BARTs, which are EBV-encoded miRNAs derived from BamH1-A rightward transcripts, are highly expressed in NPC and promote its development. A recent study showed that EBV-encoded miR-BARTS, including BART5-5p, BART7-3p, BART9-3p, and BART14-3p, downregulated the expression of a key DNA double-strand break repair gene, ataxia telangiectasia mutated (*ATM*), by targeting several sites on its 3′-UTR (27). Thus, those 4 EBV-encoded miRNAs work cooperatively to suppress *ATM* activity in response to DNA damage, contributing to NPC tumorigenesis. Those findings indicate that EBV-encoded miRNAs can be used as a novel therapeutic strategy for NPC.

## The Mechanism of miRNA Regulation in Cancers

Genes encoding miRNAs are often located at or near fragile sites and in minimal regions of loss of heterozygosity, in minimal regions of amplification, and in common cancer-related breakpoints (28). Upregulated expression of miRNAs can be caused by genomic alterations such as translocations o amplification, and loss of function can be caused by alterations such as deletions, insertions, or mutations (29). For example, the mir-17-92 cluster, which is made up of mir-17, mir-18a, mir-19b, mir-19b-1, mir-201, and mir-92-1, resides in an 800 base-pair region of the non-coding gene *MIR17HG* (also called *C13orf25*), a genomic region known to be amplified in lymphomas (30). The mir-17-92 cluster is often overexpressed in hematological cancers (31, 32). In contrast, the mir-15a-mir-16-a cluster, which resides in the chromosome 13q14 region (between exons 2 and 5 of the non-coding gene *DLEU2*), is often downregulated in patients with chronic lymphocytic leukemia due to genomic deletion of this region (31, 33).

In addition to structural genetic alterations, epigenetic modulations, including DNA promoter hypermethylation and histone hypoacetylation, have been described in solid tumors (34). For example, miR-127 is downregulated because of promoter hypermethylation in human bladder cancer (34). Usually, hypermethylation of tumor-suppressive miRNAs leads to miRNA silencing, and hypomethylation of onco-miRNAs leads to their activation and to tumorigenesis (35). In addition, long non-coding RNAs (lncRNAs) can target miRNAs, resulting in in tumorigenesis and chemo- and radioresistance. For example, lncRNA FTH1P3 promotes ATP binding cassette subfamily B member 1 (ABCB1) protein expression by targeting miR-206, acting as a miRNA “sponge,” leading to the activation of paclitaxel resistance in breast cancer (36). Circular RNAs (circRNAs) also can act as miRNA sponges to regulate miRNA expression. For example, circNT5E was recently reported to directly bind to miR-422a and inhibit its activity, promoting glioblastoma tumorigenesis (37).

The aberrant miRNA expression in cancer can also be caused by downstream miRNA processing. Merritt et al. reported that miRNA expression could be globally suppressed by short hairpin RNAs against Dicer and Drosha, 2 critical ribonucleases involved in miRNA processing (38). This miRNA suppression promotes cellular transformation and tumorigenesis.

**Figure 2:**
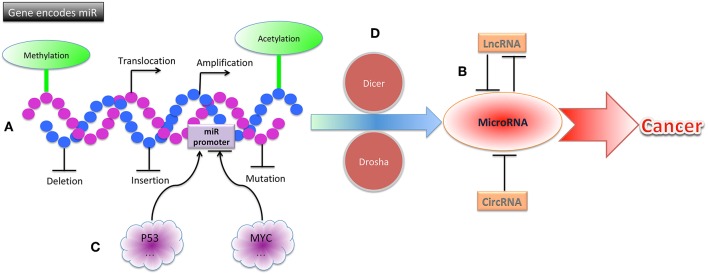
The mechanisms of miRNA dysregulation in cancers. **(A)** Structural gene alterations, including translocation, amplification, deletion, insertion, and mutation contribute to miRNA dysregulation in cancers. **(B)** Epigenetic alterations, including methylation, acetylation, lncRNAs, and circRNAs contribute to miRNA dysregulation in cancers. **(C)** MiRNA promoters can be altered by p53, MYC, and other transcription factors, leading to cancer. **(D)** Alterations of proteins involved in miRNA processing, including Dicer, Drosha, and others, can result in cancer.

The alteration of miRNA expression in cancers can also be caused by aberrant transcription factor activity, which leads to increased or decreased transcription from miRNA genes. The miR-34 miRNA family (comprising miR-34a, miR-34b, and miR-34c) is directly induced by the tumor suppressor p53. In cells with high levels of p53, miR-34 expression is elevated; furthermore, chromatin immunoprecipitation assays revealed that p53 can bind to the promoter of miR-34 (39, 40). The MYC oncoprotein downregulates transcription of tumor suppressor miRNAs such as let-7 and miR-29 family members. MYC can bind to conserved sequences of the miRNA promoter that it suppresses, and the suppression of miRNAs by MYC has been found to facilitate lymphomagenesis (41) (Figure 2).

## miRNA Dysregulation in NPC Initiation

The function of miRNAs is largely influenced by the expression of their main targets. Some miRNAs promote tumorigenesis in some cell types and suppress it in others. The classification of a miRNA as an oncogene or a tumor suppressor, therefore, requires knowledge of the type of cell in which it acts. Typically, miRNAs do not cause a specific phenotype by aiming at a single target. Instead, miRNAs target multiple mRNAs concurrently and engage in complex interactions with the machinery that controls the transcriptome. In cancers, miRNAs often are dysregulated and function collectively to mark differentiation states or individually as oncogenes or tumor suppressors. In NPC, miRNAs were reported to be expressed aberrantly and exert pivotal effects by altering the expression of their specific mRNA targets (42) (Table 1).

**Table 1:** miRNA dysregulation in NPC initiation, progression and therapies.

**miRNA**	**Target**	**Mechanism**	**Function**	**References**
miR-663	*CDKN1A*	Promotes NPC cell proliferation	Onco-miRNA	(43)
miR-125b	*TNFAIP3*	Inhibits NPC cell apoptosis	Onco-miRNA	(44)
miR-9	*CXCR4*	Suppresses NPC pathogenesis	Suppressive miRNA	(45)
miR-135a	*IL-17*	Suppresses NPC cell proliferation	Suppressive miRNA	(46)
miR-320b	*TRIAP1*	Enhances NPC cell apoptosis	Suppressive miRNA	(47)
miR-326/330-5p clusters	*CCND1*	Suppresses NPC initiation	Suppressive miRNA	(48)
miR-25	*MALAT1*	Suppresses NPC cell growth	Suppressive miRNA	(49)
Exosomal miR-23a	*TSGA10*	Promotes NPC angiogenesis	Metastasis promoter	(54)
EBV-miR-BART1/7-3p	*PTEN*	Promotes EMT process	Metastasis promoter	(55, 56)
miR-29c	*TIAM1*	Suppresses NPC metastasis	Metastasis suppressor	(57)
miR-101	*ITGA3*	Inhibits NPC angiogenesis	Metastasis suppressor	(58)
miR-203a-3p	*LASP1*	Inhibits NPC metastasis	Metastasis suppressor	(59)
miR-630	*EZH2*	Inhibits NPC cell invasion	Metastasis suppressor	(60)
EBV-miR-BART6-3p	*MIR3936HG*	Suppresses NPC metastasis	Metastasis suppressor	(61)
miR-324-3p	*WNT2B*	Reduces NPC radioresistance	Radiosensitizer	(69)
miR-519d	*PDRG1*	Sensitizes NPC to IR	Radiosensitizer	(70)
miR-24	*COPS5/SP1*	Enhances NPC radiosensitivity	Radiosensitizer	(15)
miR-19b-3p	*TNFAIP3*	Increases NPC radioresistance	Radioresistant agent	(63)
miR-3188	*mTOR*	Suppresses NPC resistance of 5-FU	Chemosensitizer	(67)

*CCND1, cyclin D1; CDKN1A, cyclin-dependent kinase inhibitor 1A; CXCR4, C-X-C motif chemokine receptor 4; IL-17, interleukin-17; MALAT1, metastasis-associated lung adenocarcinoma transcript 1; TNFAIP3, tumor necrosis factor alpha-induced protein 3; TRIAP1, TP53-regulated inhibitor of apoptosis 1; CXCL12, C-X-C motif chemokine ligand 12; EZH2, enhancer of zeste homolog 2; ITGA3, integrin subunit alpha 3; LASP1, LIM and SH3 protein 1; MIR3936HG, a long noncoding RNA; PTEN, phosphatase and tensin homolog; TIAM1, T cell lymphoma invasion and metastasis 1; TSGA10, testis-specific 10; 5-FU, fluorouracil; COPS5, COP9 signalosome subunit 5 (also called JAB1); MTOR, mechanistic target of rapamycin kinase; PDRG1, p53 and DNA damage regulated 1; SP1, specificity protein 1; WNT2B, Wnt family member 2B*.

### Onco-miRNAs in NPC

Tumor suppressor genes are usually inhibited by miRNAs directly, and such miRNAs are considered to be onco-miRNAs. For example, cell cycle inhibitor *CDKN1A* (also called *P21*) is believed to be directly targeted by miR-663 in NPC, leading to the promotion of NPC cell proliferation and tumorigenesis (43). An onco-miRNA, miR-125b, was found to be significantly upregulated in the NPC tissue compared with healthy nasopharyngeal mucosa, and this upregulation was correlated with poor survival outcomes; furthermore, high expression of miR-125b was identified as an independent predictor for shorter survival durations in NPC patients (44). The same study found that miR-125b promoted proliferation and inhibited apoptosis in NPC cells. A direct target of miR-125b, the tumor necrosis factor alpha-induced protein 3 gene *TNFAIP3* (formerly called *A20*), functions as a tumor suppressor in NPC and mediates miR-125b-promoted NPC tumorigenesis by activating the nuclear factor κB (NF-κB) signaling pathway. Together, these findings demonstrate that onco-miRNAs target tumor suppressor genes, leading to NPC initiation.

### Tumor-Suppressive miRNAs in NPC

Oncogenes can be suppressed by miRNAs directly, and such miRNAs are considered to be tumor-suppressive miRNAs. For example, the C-X-C motif chemokine receptor 4 oncogene *CXCR4* can be directly targeted by tumor-suppressive miR-9, resulting in the inhibition of NPC pathogenesis. In NPC clinical specimens, miR-9 was observed to be downregulated (45). Interleukin-17 (IL-17), a proinflammatory cytokine, suppresses immune defense, and immune surveillance while promoting tumor growth. A study showed that IL-17 was targeted by miR-135a, resulting in the inhibition of NPC cell proliferation (46). In another study, overexpression of miR-320b was shown to suppress NPC cell proliferation and enhance mitochondrial fragmentation and apoptosis (47). In contrast, silencing miR-320b enhanced NPC tumor growth and inhibited cell apoptosis. The TP53-regulated inhibitor of apoptosis 1 gene, *TRIAP1*, has been found to be directly targeted by miR-320b, which mediates *TRIAP1*'s role in NPC cell proliferation inhibition and apoptosis induction. It has also been reported that miR-326/330-5p clusters can target cyclin D1 gene, *CCND1*, exerting their tumor-suppressive roles on NPC initiation (48). Some tumor-suppressive miRNAs target lncRNAs that function as oncogenes. For example, miR-25 expression was found to be upregulated in NPC cells, and its ectopic expression was shown to suppress NPC cell growth and motility by targeting metastasis-associated lung adenocarcinoma transcript-1 (*MALAT1*), a proto-oncogenic lncRNA (49). The tumor-suppressive miRNAs miR- 451 and miR-539-5p inhibit NPC initiation by targeting the macrophage migration inhibitory factor (*MIF*) and Kruppel-like factor 12 (*KLF12*) genes, respectively (50, 51). Finally, lentivirus can be used as a delivery system to overexpress specific tumor-suppressive miRNAs in NPC, resulting in the inhibition of NPC initiation. For example, lenti-miR-26a was shown to inhibit the tumorigenicity of NPC cells in nude mice significantly, providing a useful strategy for treating NPC patients (52).

## miRNA Dysregulation in NPC Progression

NPC is an aggressive disease that tends to spread locally and metastasize to regional lymph nodes and distant organs. Distant metastasis is the principal mode of treatment failure (53). It is known that NPC metastasis is associated with miRNA dysregulation. The functions of miRNAs often are contradictory because they are determined by the cellular environment and the stage of the metastatic process. Therefore, identifying which miRNAs promote or suppress the metastatic process of NPC could lead to the development of new, efficient therapeutic agents to prevent or delay metastasis (Table 1).

### Metastasis Promoter miRNAs in NPC

Mounting evidence implicates miRNAs in the modulation of angiogenesis, which is essential to the metastatic process. For instance, it has been reported that exosomal miR-23a overexpression promotes angiogenesis in NPC by directly targeting the testis-specific 10 gene, *TSGA10* (54). Furthermore, miR-23a overexpression in pre-metastatic NPC tissue was identified as a prognostic biomarker for early metastasis. In addition, EBV-encoded miR-BART1 has been shown to induce NPC metastasis by regulating pathways that depend on the phosphatase and tensin homolog gene, *PTEN* (55). Another EBV-encoded miRNA, miR-BART7-3p, was shown to promote the epithelial-mesenchymal transition (EMT) and metastasis of NPC cells by suppressing PTEN and consequently activating the PI3K/AKT/GSK-3β signaling pathway (56).

### Metastasis Suppressor miRNAs in NPC

The tumor-suppressive miR-29c is also a metastasis suppressor that inhibits NPC cell migration, invasion, and metastasis by targeting the T cell lymphoma invasion and metastasis 1 gene, *TIAM1*, directly (57). In NPC patient samples and cell lines, miR-101 was found to be downregulated, and its ectopic expression significantly inhibited cell migration, invasion, and angiogenesis both *in vitro* and *in vivo*. The prometastatic gene integrin subunit alpha 3 (*ITGA3*) has been identified and validated as a target of miR-101 and shown to mediate the suppressive effects of miR-101 on NPC metastasis. Interestingly, the systemic delivery of lentivirus-mediated miR-101 in NPC suppressed lung metastatic colony formation with no noticeable toxic effects (58). Also, miR-203a-3p was found to be dysregulated and to act as a tumor suppressor in NPC. This miRNA suppresses NPC metastasis by targeting the LIM and SH3 protein 1 gene, *LASP1* (59).

Metastasis suppressor miRNAs can also target or be targeted by lncRNAs in NPC. For example, lncRNA *H19* has been found to be overexpressed in NPC tissue, and *H19* knockdown significantly suppressed invasion NPC cells. *H19* knockdown downregulated the expression of the enhancer of zeste homolog 2 gene, *EZH2*, which is upregulated in NPC and promotes invasion. *H19* does not bind directly to *EZH2* but instead modulates its expression by suppressing the activity of miR-630, which inhibits *EZH2* and interacts with *H19* in a sequence-specific manner. *H19* also suppresses E-cadherin expression and promotes invasion in NPC cells through the miR-630/EZH2 pathway (60). And He et al. demonstrated that EBV-miR-BART6-3p suppressed EBV-associated cancer cell migration and invasion by targeting lncRNA *MIR3936HG* (also known as *LOC553103*) and reversing the EMT process. And *MIR3936HG* knockdown by specific siRNAs was shown to phenocopy the effect of EBV-miR-BART6-3p, while elevated *MIR3936HG* expression enhanced tumor cell migration and invasion to promote EMT (61).

## miRNA Dysregulation-Related Signaling Pathways in NPC

Several signaling pathways are involved in miRNA dysregulation-related processes in NPC. For example, miR-125b was shown to promote NPC tumorigenesis by activating the NF-κB signaling pathway, which plays a critical role in NPC tumorigenesis and progression (44, 62). In addition, miR-19b-3p was found to be upregulated and to be an independent predictor for poor survival outcomes in NPC patients. MiR-19b-3b increased NPC cell radioresistance by targeting *TNFAIP3* and then activating the NF-κB signaling pathway (63).

The PTEN/AKT pathway plays an important role in NPC processes related to miRNA dysregulation. One study found that miR-141 was markedly elevated in NPC tissues and negatively correlated with both patient survival and the expression of the bromodomain containing 7 gene, *BRD7; BRD7* overexpression activated the PTEN/AKT pathway, but restoring miR-141 expression suppressed this activation and partially restored NPC cell proliferation and tumor growth. The BRD7/miR-141/PTEN/AKT axis therefore is important to NPC progression and could provide new treatment targets and diagnostic markers (64). In addition, EBV-encoded miRNAs miR-BART1 and miR-BART7-3p promote NPC metastasis by modulating the PTEN/PI3K/AKT signaling pathway (55, 56). PI3K signaling is also involved in miRNA dysregulation-related processes in NPC. A study of NPC tumor specimens found that tumor-suppressing protein PDCD4 suppresses the pPI3K/pAKT/c-JUN signaling pathway, which in turn modulates miR-374a's binding to *CCND1*, resulting in dysregulation of NPC cell growth, metastasis, and chemoresistance (65). In this study, miR-374a expression was positively correlated with PDCD4 expression and negatively correlated with *CCND1* expression. The PI3K/AKT/mTOR signaling pathway also significantly affects NPC tumorigenesis and development (66). For example, miR-3188 was shown to inhibit NPC cell cycle transition and proliferation, to sensitize cells to chemotherapy, and to extend survival in tumor-bearing mice, and to inactivate p-PI3K/p-AKT/c-JUN signaling by targeting mTOR directly, further suppressing the cell cycle through the p-PI3K/p-AKT/p-mTOR pathway (67).

## miRNA Dysregulation in NPC Therapies

Radiotherapy and chemotherapy are 2 main treatments for NPC. Mounting evidence shows that miRNAs are dysregulated during radio- or chemotherapy for NPC and may reduce or induce the sensitivity of NPC cells to radiotherapy or chemotherapy (Table 1).

### miRNA Dysregulation in Radiotherapy

Radioresistance is the main reason for NPC treatment failure (68). Multiple studies have shown that miRNA expression in various cell types changed upon irradiation, as did the specific effects of various miRNAs on cellular radiosensitivity. It has been reported that miR-324-3p reduces NPC radioresistance by directly targeting the well-known oncogene Wnt family member 2B (*WNT2B*), inhibiting the gene's translation (69). Studies have also reported that miR-519d sensitizes NPC cells to radiation by directly targeting the 3′-UTR of *PDRG1* (p53 and DNA damage regulated 1) mRNA (70) and thatiR-24 increases radiosensitivity in NPC by targeting both *COPS5* and *SP1* (specificity protein 1) (15). In contrast, Huang et al. demonstrated that miR-19b-3p upregulation decreases—and downregulation increases—NPC sensitivity to radiation. The researchers also found that miR-19b-3p directly targeted *TNFAIP3*, and the gene's upregulation reversed miR-19b-3p's suppressive effects on NPC cell radiosensitivity. Thus, miR-19b-3p was shown to enhance radioresistance in NPC cells by activating the TNFAIP3/ NF-κB pathway (63). Together, these studies indicate the potential use of miRNAs as radiosensitizing agents in NPC treatment.

### miRNAs Dysregulation in Chemotherapy

The importance of miRNAs in chemotherapy response has been demonstrated in multiple human cancers, including cancer of the tongue (71). In NPC, miR-3188 has been found to inhibit cell growth and resistance to fluorouracil by directly targeting the mechanistic target of rapamycin kinase gene, *MTOR*, and regulating the cell cycle (67). Another study showed that the metastasis suppressor miR-29c can also increase NPC cells' sensitivity to both radiotherapy and cisplatin-based chemotherapy (72). The above evidence shows that miRNAs mainly function as chemosensitizers in NPC.

## miRNAs as Biomarkers and Novel Therapeutic Approaches in NPC

In the above sections, we showed that miRNAs are dysregulated during NPC initiation, progression, and therapy. In addition, several studies have reported that miRNA dysregulation is associated with the survival of NPC patients, and miRNAs may serve as independent biomarkers for NPC diagnosis, recurrence, and prognosis. Furthermore, a few molecularly targeted drugs have emerged as clinically active against advanced NPC in recent years (73), and the exploration of miRNAs as drugs or drug targets against other cancer types is already underway (29).

### miRNAs as Biomarkers in NPC

Several miRNAs show potential as biomarkers in NPC. A recent meta-analysis indicated that increased miRNA expression led to a poor overall survival and increased the likelihood of death of NPC patients (74). The tumor and metastasis suppressor miR-29c has been shown to be downregulated in both the serum and tumor tissue of NPC patients, indicating its promise as a biomarker for NPC diagnosis, prognosis, and recurrence (75). Also, NPC patients were shown to have significantly higher serum levels of miR-663 compared with healthy individuals, and high levels were associated with worse 5-year overall and relapse-free survival outcomes in NPC patients (76). In addition, chemotherapy significantly lowered NPC patients' serum miR-663 levels. These results suggest a critical role for miR-663 as a biomarker of NPC prognosis and response to chemotherapy. Recent studies showed that miR-31-5p was downregulated in present in NPC tissues and cell lines, acting as a tumor suppressive miRNA. And circulating miR-31-5p was identified to be a potential novel and non-invasive biomarker for the early diagnosis of NPC (77). The expression levels of tumor-educated platelet miR-34c-3p and miR-18a-5p are upregulated in NPC, which are promising novel liquid biopsy biomarkers for NPC diagnosis (78). In addition, miR-342-3p was significantly downregulated in NPC specimens and its low expression was significantly correlated with reduced overall survival of NPC patients, indicating miR-342-3p as a biomarker of NPC prognosis (79). All in all, the above results suggest miRNA can be a single biomarker for NPC diagnosis, prognosis, and response to therapy.

Additionally, miRNA signatures have been more and more identified to be novel biomarkers of NPC prognosis and prediction. For example, a two-miRNA signature of miR-548q and miR-483-5p were identified as potential biomarkers of NPC by comparing the plasma miRNA profiles of 31 NPC patients and 19 controls (80). And a three-miRNA signature including miR-548q, miR-630, and miR940 were increased in the plasma of NPC patients compared to those of controls. They are potential novel and useful biomarkers for NPC detection and diagnosis (81). The plasma level of miR-483-5p, miR-103, and miR-29a could be helpful to predict survival in patients with NPC (82). A four-miRNA signature of miR-22, miR-572, miR-638, and miR-1234 were identified to be prognostic biomarkers of NPC to the TNM staging system (83). Recently, a 8-miRNA signature including miR-188-5p, miR-1908, miR-3196, miR-3935, miR-4284, miR-4433-5p, miR-4665-3p, miR-513b, and 16-miRNA signature including miR-1224-3p, miR-1280, miR-155-5p, miR-1908, miR-1973, miR-296-5p, miR-361-3p, miR-425-5p, miR-4284, miR-4436b-5p, miR-4439, miR-4665-3p, miR-4706, miR-4740-3p, miR-5091, miR-513b are promising biomarkers for NPC diagnosis (84). Taker together, miRNAs can be as both single biomarkers and signatures for NPC detection, diagnosis, and prognosis (Table 2).

**Table 2:** miRNAs as biomarkers in NPC.

**MiR**	**Expression in NPC**	**Biomarker**	**References**
MiR-29c	Downregulated in NPC tissue	NPC diagnosis and prognosis	(75)
MiR-663	Upregualted in NPC serum	NPC prognosis and response to chemotherapy	(76)
MiR-31-5p	Downregulated in NPC tissue	NPC diagnosis	(77)
MiR-34c-3p	Upregualted in NPC tissue	NPC diagnosis	(78)
MiR-18a-5p	Upregualted in NPC tissue	NPC diagnosis	(78)
MiR-342-3p	Downregulated in NPC tissue	NPC prognosis	(79)
**MiR signatures**			
2-miRNA: miR-548q and miR-483-5p		NPC detection	(80)
3-miRNA: miR-548q, miR-630, and miR940		NPC detection and diagnosis	(81)
3-miRNA: miR-483-5p, miR-103, and miR-29a		NPC prognosis	(82)
4-miRNA: miR-22, miR-572, miR-638, and miR-1234		NPC prognosis	(83)
8-miRNA: miR-188-5p, miR-1908, miR-3196, miR-3935, miR-4284, miR-4433-5p, miR-4665-3p, miR-513b		NPC diagnosis	(84)
16-miRNA: miR-1224-3p, miR-1280, miR-155-5p, miR-1908, miR-1973, miR-296-5p, miR-361-3p, miR-425-5p, miR-4284, miR-4436b-5p, miR-4439, miR-4665-3p, miR-4706, miR-4740-3p, miR-5091, miR-513b		NPC diagnosis	(84)

### miRNAs as Therapeutic Approaches in NPC

One of the most appealing properties of miRNAs as therapeutic agents is their ability to simultaneously target more than 1 gene, making miRNAs extremely efficient for regulating distinct cell processes relevant to normal and malignant cell homeostasis. In NPC, miRNAs for gene therapy have been delivered using lentiviral vectors. For example, a previous study found that tumor suppressor miR-31-5pinhibited EBV-positive NPC tumorigenesis, and minicircle-oriP-miR-31, a novel EBNA1-specific miRNA delivery system, was constructed and shown to inhibit NPC cell proliferation and migration *in vitro* and to suppress xenograft growth and lung metastasis *in vivo*. The researchers also found that the WD repeat domain 5 gene, *WDR5*, is a target of miR-31-5p. The study proved that targeted delivery of miR-31-5p using a non-viral minicircle vector could serve as a novel therapeutic approach for NPC, indicating a promising miRNA therapy for NPC patients (85). More studies are still ongoing to apply miRNA therapy to patients with NPC.

## Conclusions and Future Directions

The need for novel therapeutic targets and agents for treating NPC patients is urgent, owing to NPC's anatomical location and resistance to both radiotherapy and chemotherapy. Large numbers of miRNAs are dysregulated during the process of NPC initiation, progression, and therapy; therefore, miRNAs have been proposed as useful biomarkers to predict prognosis in and therapeutic approaches to cure patients with NPC. The advantages of using miRNAs as drugs antagonizing NPC is that 1 miRNA can target multiple targets and the same target can be targeted by many miRNAs, showing their comprehensive potential roles in the clinic. Based on the previous studies on miRNA dysregulations in NPC, how to use and take the advantages of miRNAs in clinic is to be solved. And due to the fact that miRNAs can regulate many mRNAs, the potential of toxic phenotypes and other off-target effects of miRNA treatment approaches is a major concern. As a result, more and more studies focusing on the toxic effects of targeting miRNAs are required before such therapies can be used safely in NPC patients.

## Author Contributions

SW was responsible for the writing and editing of the manuscript. F-XC was responsible for modifying the manuscript. WW provided some critical useful suggestions.

### Conflict of Interest Statement

The authors declare that the research was conducted in the absence of any commercial or financial relationships that could be construed as a potential conflict of interest.

## Abbreviations

A20/TNFAIP3: tumor necrosis factor alpha-induced protein 3
ABCB1: ATP binding cassette subfamily B member 1
ATM: ataxia telangiectasia mutated
BRD7: bromodomain containing 7
CAPN4/CAPNS1: calpain small subunit 1
circRNA: circular RNA
COPS5: COP9 signalosome subunit 5
CXCL12: C-X-C motif chemokine ligand 12
CXCR4: C-X-C motif chemokine receptor 4
DGCR8: Drosha and DiGeorge syndrome critical region gene 8
E2F3: E2F transcription factor 3
EBV: Epstein-Barr virus
EMT: epithelial-mesenchymal transition
EZH2: enhancer of zeste homolog 2
*FGF5*: fibroblast growth factor 5
IL-17: interleukin-17
ITGA3: integrin subunit alpha 3
KLF12: Kruppel-like factor 12
LASP1: LIM and SH3 protein 1
lncRNA: long non-coding RNA
MALAT1: metastasis-associated lung adenocarcinoma transcript-1
AP3K5/ASK1: apoptosis signal-regulating kinase1
miRNA: microRNA
MIF: macrophage migration inhibitory factor
mTOR: mechanistic target of rapamycin kinase
NF-κB: nuclear factor κB
NPC: nasopharyngeal carcinoma
PDRG1: p53 and DNA damage regulated 1
PTEN: phosphatase and tensin homolog
RISC: RNA-induced silencing complex
RP: ribosomal protein
SP1: specificity protein 1
TIAM1: T cell lymphoma invasion and metastasis 1
TRIAP1: TP53-regulated inhibitor of apoptosis 1
TSGA10: testis-specific gene antigen 10
UTR: untranslated region
WNT2B: Wnt family member 2B
XIST: X inactivate-specific transcript.
